# A Screen for Selective Killing of Cells with Chromosomal Instability Induced by a Spindle Checkpoint Defect

**DOI:** 10.1371/journal.pone.0047447

**Published:** 2012-10-15

**Authors:** Zeeshan Shaukat, Heidi W. S. Wong, Shannon Nicolson, Robert B. Saint, Stephen L. Gregory

**Affiliations:** 1 School of Molecular and Biomedical Sciences, University of Adelaide, Adelaide, South Australia, Australia; 2 Department of Genetics, University of Melbourne, Melbourne, Victoria, Australia; University College London, United Kingdom

## Abstract

**Background:**

The spindle assembly checkpoint is crucial for the maintenance of a stable chromosome number. Defects in the checkpoint lead to Chromosomal INstability (CIN), which is linked to the progression of tumors with poor clinical outcomes such as drug resistance and metastasis. As CIN is not found in normal cells, it offers a cancer-specific target for therapy, which may be particularly valuable because CIN is common in advanced tumours that are resistant to conventional therapy.

**Principal Findings:**

Here we identify genes that are required for the viability of cells with a CIN phenotype. We have used RNAi knockdown of the spindle assembly checkpoint to induce CIN in *Drosophila* and then screened the set of kinase and phosphatase genes by RNAi knockdown to identify those that induce apoptosis only in the CIN cells. Genes identified include those involved in JNK signaling pathways and mitotic cytoskeletal regulation.

**Conclusions/Significance:**

The screen demonstrates that it is feasible to selectively kill cells with CIN induced by spindle checkpoint defects. It has identified candidates that are currently being pursued as cancer therapy targets (e.g. Nek2: NIMA related kinase 2), confirming that the screen is able to identify promising drug targets of clinical significance. In addition, several other candidates were identified that have no previous connection with mitosis or apoptosis. Further screening and detailed characterization of the candidates could potentially lead to the therapies that specifically target advanced cancers that exhibit CIN.

## Introduction

Chromosomal INstability (CIN) is a common feature of nearly all solid tumors [1]. CIN results in ongoing numerical and structural aberrations of chromosomes as tumors proliferate, and is associated with poor clinical outcomes such as tumour metastasis and adaptability to environmental and chemical stresses [2], [3]. Drug resistance and relapse is common in cancers with CIN as they evolve rapidly, making them difficult to target with regular therapies [4], [5].

The most common errors seen in CIN cancer cells are lagging chromosomes and chromosomal bridges. The mechanisms proposed to be responsible for these errors include defects in: sister chromatid cohesion [6], kinetochore–spindle attachment [7], cytokinesis [8], and centrosome duplication [9]. Perhaps the best-characterized cause of chromosomal instability is weakening of the Spindle Assembly Checkpoint (SAC) [10], [11].

The SAC is the only mechanism by which cells can detect aberrant kinetochore attachments in metaphase and delay the entry to anaphase until the problem is resolved, or otherwise induce apoptosis [12], [13]. This mechanism is not perfect, so mutations that cause high rates of segregation defects or shorten the duration of the metaphase error checking can cause CIN and lead to tumorigenesis [10], [11]. The list of mutations known to have effects on chromosomal segregation is long, and includes over- and under-expression of spindle checkpoint proteins and clinically relevant cancer mutations such as loss of Rb in retinoblastoma or Adenomatous Polyposis Coli (APC) in colorectal cancer [11], [14]. Reduction of the SAC protein Mad2 (mitotic arrest deficient 2) or its partner proteins (e.g. BubR1: Budding uninhibited by benzimidazoles Related 1) have been shown to shorten metaphase, causing CIN, aneuploidy and tumour susceptibility in humans and mouse models [15]–[17]. However, even in those CIN cancers that retain a SAC capable of detecting gross spindle abnormalities [18], the checkpoint is not able to respond to the merotelic kinetochore attachments that cause instability [19]. Loss of function of p53, which is common in cancers [20], increases the tolerance level for such missegregation, allowing the continual reassortment of the genome seen in CIN tumours [21], [22]. CIN levels are higher in malignant tumors than in benign ones [23], [24] and CIN is not found in normal cells, so it offers an attractive target for a cancer-specific therapy. CIN is particularly common in tumour types that are most in need of new drugs (e.g. colorectal cancers). Targeting CIN could also help to limit the ability of cancer cells to evolve drug resistance and other poor clinical outcomes, which may increase the efficacy of current therapies.

Here we have used depletion of the SAC to induce CIN. We have then carried out a systematic genome-wide screen for kinase and phosphatase genes that, when depleted, can trigger apoptosis only in these genetically unstable cells, but not in normal cells. Our rationale is that for a therapy to be effective, cell death should be restricted to the tumour, i.e. the cells that exhibit CIN. We set up an assay system using *Drosophila melanogaster* in which we induced tissue-specific chromosomal instability in a wild type organism. We generated CIN by knocking down the SAC protein Mad2, which shortens metaphase, giving cells less time to correctly orient their chromosomes before the onset of anaphase [25], resulting in chromosomal bridges and lagging chromosomes. While there may be numerous defects that lead to CIN in a tumour, loss of Mad2 is found as a contributing factor in a range of CIN cancers [26], [27] and more than 80% of colorectal cancers carry APC mutations that have been shown to sequester Mad2 [28] and BubR1 [29] at least in some cell lines [30].

Kinases and phosphatases are key regulatory enzymes controlling vital processes such as cell growth, differentiation, and survival [31]. Alteration in levels of these proteins can lead to abnormal cell growth and cell death mechanisms, which can result in tumorigenesis [32]. Many kinases and phosphatases are approved as good drug targets and currently they are the main focus of drug discovery efforts against cancer [33]. Our screening of kinase and phosphatase genes in a CIN background gave a set of potential candidates that reproducibly caused significant lethality via apoptosis in CIN flies compared to their non-CIN control siblings. The screen identified several groups of candidates including centrosomal proteins such as Nek2, which is currently being pursued as a therapeutic target for cancer [34]. These results may contribute to the identification of novel targets that can specifically kill advanced, drug resistant, CIN tumor cells without harming normal cells.

## Results

### Screening for Candidate Knockdowns that Kill Cells with SAC-induced Chromosomal Instability

**Figure 1:**
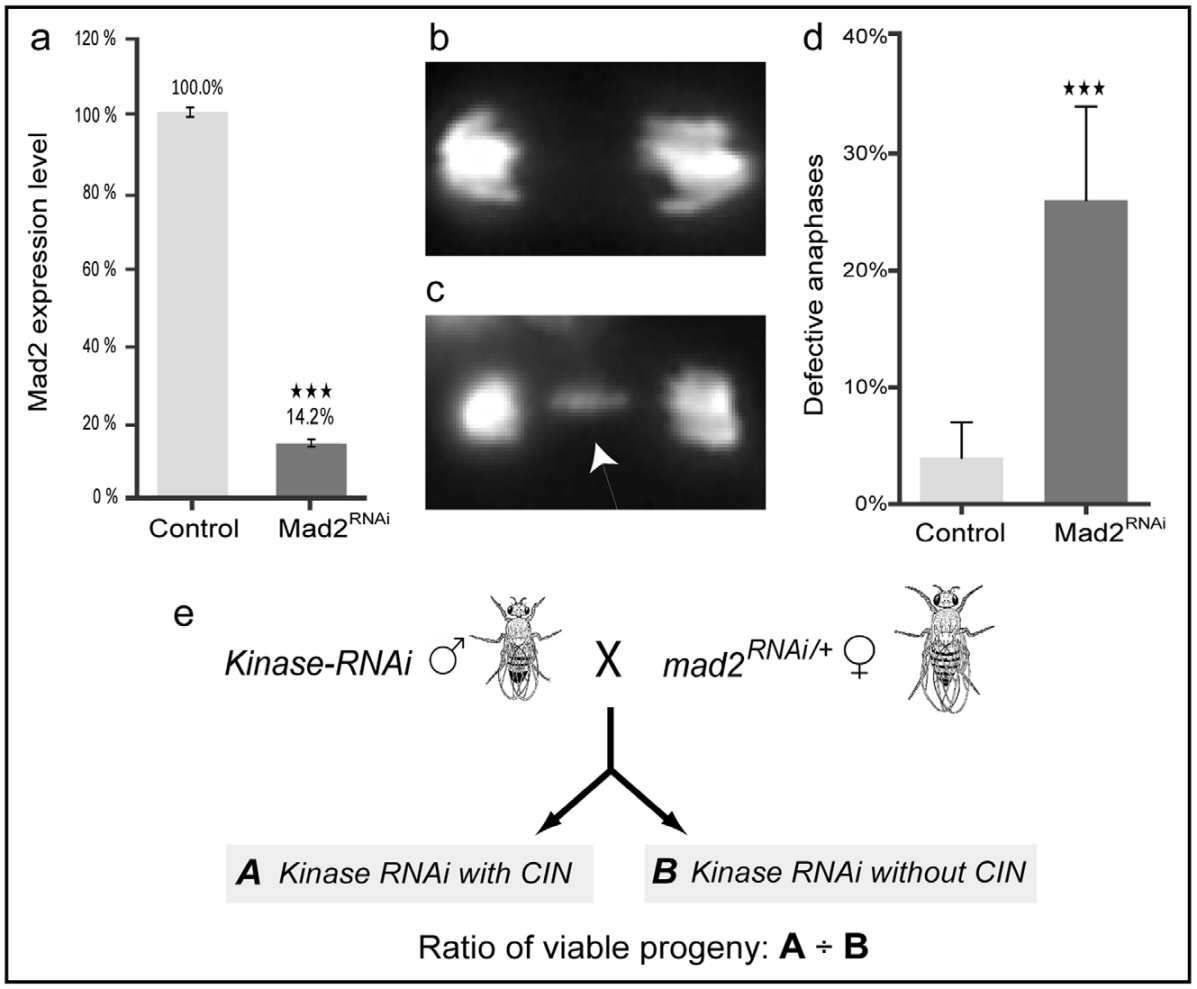
Establishment of a screening strategy using an induced-CIN model. (a) Reverse transcriptase-qPCR shows that the ubiquitous expression of UAS-*mad2* RNAi resulted in ∼85% knocked down of *mad2* expression level (black bar) which is significantly less than the mad2 level in UAS-LacZ RNAi control (grey bar). Error bars represent SD. P-values are calculated by two-tailed Student’s t-test: p<0.001 = ★★★. (b–c) Third instar larval brain cells stained with Hoechst 33342 to label DNA. (b) Normal segregation in a wild type anaphase. (c) Defective anaphase in an induced-CIN brain cell (*da>mad2*) resulting in a lagging chromosome (arrowed). (d) The fraction of defective anaphases (lagging chromosomes or bridges) observed in *mad2* knocked down (black bar) brain squashes and wild type controls (grey bar). Error bars represent 95% CIs. P-values are calculated by two-tailed Fisher’s exact test: p<0.001 = ★★★. (e) Diagrammatic representation of viability screen crosses. Males with *Kinase-RNAi* (UAS-kinase^dsRNA^) were crossed with females carrying the CIN background (UAS-*mad2^dsRNA^*; *da-Gal4*). Progeny were double knockdown (A: *mad2* and *kinase*) or single knockdown (B: *kinase* only). The ratio of viable progeny A/B was used to rank candidates for further analysis.

To generate a model system in which we could induce chromosomal instability we expressed dsRNA to knock down Mad2 and thereby weaken the SAC in *Drosophila*. Ubiquitous expression of *mad2* dsRNA in the whole organism gives ∼85% reduction of Mad2 expression as compared to control *lacZ* dsRNA (Figure 1a). This depletion of Mad2 resulted in >25% of anaphases showing lagging chromosomes or chromosomal bridges in larval brains (Figure 1b–d), without compromising the overall viability of the organism. This survival, despite a significant rate of anaphase errors, is consistent with the viable amorphic *mad2* allele described by Buffin et al. [25]. We used this background to screen a set of gene knockdowns to identify candidates that could specifically kill cells with CIN but not normal dividing cells. We screened the *Drosophila* kinome, testing knockdown of 397 kinase and phosphatase genes for those that were lethal only in a CIN background (Figure 1e). This screening ranked the set of genes to identify those that when depleted in the whole organism, reproducibly caused the most lethality in CIN flies compared to their non-CIN control siblings (Table S1). The siblings vary only in whether or not they have induced CIN, so a deviation from the expected 1∶1 ratio of CIN: non-CIN progeny indicated a CIN-specific effect of the candidate on viability. We observed the whole range of responses from complete lethality in a CIN background through to no effect, and we prioritized those candidates with the strongest CIN-specific lethality.

### Screening for Cell Death

We investigated the cellular phenotypes of 26 kinases from the initial screen that gave more than 75% lethality in a CIN background, that is, more than four non-CIN for every one surviving CIN sibling. This lethality could have resulted from developmental or patterning failures, so we wished to test whether our candidates generated cell death in CIN cells. First, we examined the effect on wing development when candidates were depleted with or without *mad2* (Figure S1a), and these data were quantified by measuring the amount of tissue loss (notching) in the affected wing area (Figure S1b). 17 candidates resulted in significant cell loss in adult wings when Mad2 was reduced as compared to controls (Table 1). The most promising candidates identified from this screening included genes from some well characterized groups such as those involved in JNK (Jun N-terminal kinase) signaling and centrosomal regulation as well as others (e.g. PAS Kinase) with no previous connection to cell division.

**Table 1:** Candidates giving CIN-dependent cell death.

Groups	Candidate symbol	Mammalian homolog	Functional association
Centrosomal	*Nek2*	NIMA-related kinase 2 (NEK2)	Cell cycle progression [61]
	*lok*	checkpoint homolog (Chk2)	DNA damage [62]
	*asp*	Abnormal spindle (Aspm)	Spindle organization [47]
	*tefu*	Ataxia telangiectasia mutated (ATM)	DNA damage response [62]
	*bsk*	JUN amino terminal kinase (JNK)	JNK signaling pathway [63]
JNK pathway	*bsk*	JUN amino terminal kinase (JNK)	JNK signaling pathway [63]
	*pvr*	PDGF/VEGF receptor	JNK activator [64]
	*slpr*	JUN kinase kinase kinase (JNKKK)	JNK signaling pathway [65]
	*Pak3*	p21 protein (Cdc42/Rac)-activated kinase 3 (PAK3)	JNK activator [66]
DNA damage	*lok*	checkpoint homolog (Chk2)	DNA damage [62]
	*tefu*	Ataxia telangiectasia mutated (ATM)	DNA damage response [62]
Wnt signaling pathway	*mbt*	p21 protein (Cdc42/Rac)-activated kinase 4 (PAK4)	Wnt signaling/cytoskeletal regulation [67]
	*aPKC*	Protein kinase C	Wnt signaling [68]
	*Wnk*	WNK lysine deficient protein kinase 1 (WNK1)	Ion regulation, cell cycle progression and adaptation [69]
Histone kinases	*CG8878*	vaccinia-related kinase (VRK)?	Histone kinase?
	*ball*	Nucleosomal histone kinase-1 (Nhk-1)	Histone kinase [70]
Others	*Pink1*	PTEN-induced putative kinase 1	apoptosis/mitophagy [71]
	*lic*	mitogen-activated protein kinase kinase 3 (MAP2K3)	MAP kinase-mediated signaling [72]
	*CG4041*	TBC1 domain containing kinase (TBCK)/Rab gtpase?	Unknown
	*Pask*	PAS kinase (PASKIN)	cellular energy homeostasis [52], [53]

Candidates from the viability screening and cell death assay that gave the most CIN-dependent cell death. Some of the candidates are placed in more than one group on the basis of their associations.

**Figure 2:**
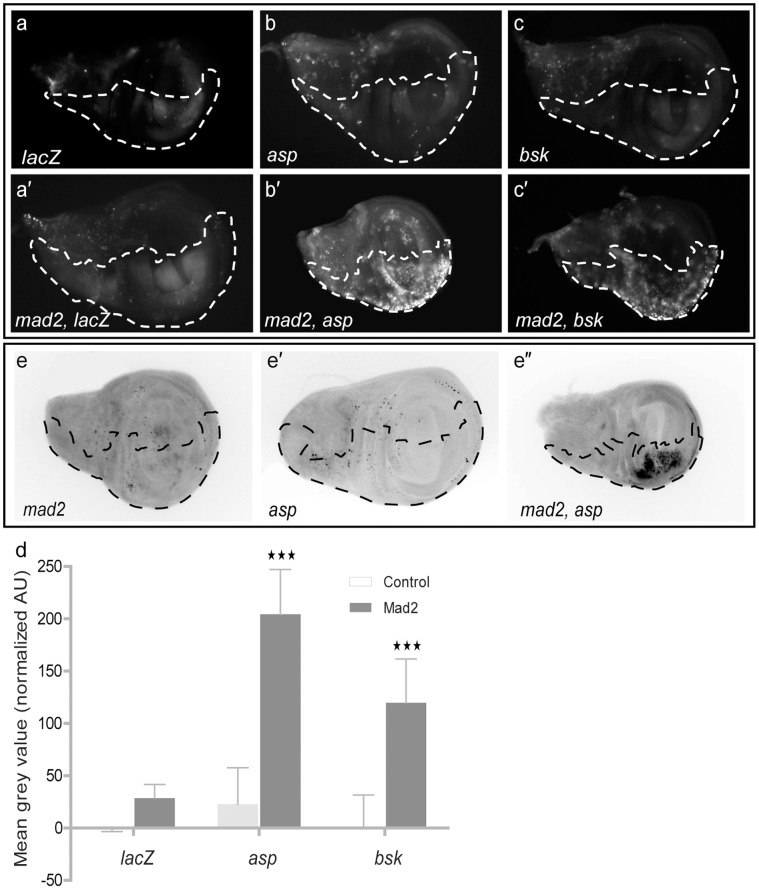
Cell death assays on larval wing discs. Dotted line shows the *en-CD8GFP* marked compartment or tester region in which genes were depleted. The other half of each wing disc expressed no transgenes and serves as an internal control. (a–c, a′–c′) Images of wing discs stained with Acridine Orange to show cell death. (a) Negative control (*lacZ RNAi*), (a′) *LacZ* and *mad2* RNAi, (b & c) Candidate RNAi (*asp* and *bsk*), (b′ & c′) double knockdown of candidate and Mad2. (d) Graph shows quantitation of Acridine Orange staining (above wild type) in control and candidate imaginal wing disc halves with or without *mad2* RNAi. Error bars represent 95% CIs, n≥8 in all cases. P-values were calculated by two-tailed t-tests with Welch’s correction: p<0.001 = ★★★. (e-e′) Cleaved caspase 3 staining showing apoptosis in e: *mad2 RNAi,* e′: *asp RNAi* and double knockdown (e″: *asp RNAi* and *mad2 RNAi*).

Acridine Orange staining of larval wing discs showed significantly elevated levels of cell death when the candidates were knocked down in CIN cells (e.g. Figure 2), consistent with the wing tissue loss being caused by cell death in the affected region. Quantification of the Acridine Orange stains was carried out by measuring the average signal per unit area in each half of the disc and subtracting the background value (from the wild type, control half) from the RNAi affected half, to show the effect of the knockdown. This gave data consistent with the adult wing tissue loss results (complete data shown in Figures S2a and S2b). Note that loss of Mad2 by itself gave little cell death (Figure 2a’; [25]), as did depletion of the candidates alone (Figure 2b, 2c), consistent with these candidates only being required for the survival of genetically unstable cells. To validate our model we induced CIN by knocking down another SAC protein, BubR1 [35], and found that our candidates also induced cell death in this CIN background (e.g. Figure S4). Interestingly, not all candidates showing a high level of cell death in a CIN background were completely depleted by the candidate-RNAi. For example, *bsk* and *asp* knockdown still left 46% (±4%) and 63% (±3%) respectively of the wild type RNA levels when measured by qPCR. This partial knockdown was expected for essential genes like *bsk* and *asp,* because our original screen selected for candidate RNAi lines that were not lethal in normal cells. CIN cells must be highly sensitive to dosage variations in these candidates to give such strong phenotypes following modest candidate depletion, emphasizing the significant role these candidates have in cellular responses to CIN.

### Apoptosis in CIN Cells

To confirm that the cell death observed was a result of the activation of apoptosis, we used antibody staining for the active form of effector caspase 3 (Figure 2e). Consistent with the results of Acridine Orange staining, knockdown of either Mad2 or a candidate alone showed little apoptosis-specific cleaved caspase 3 staining. However when we knocked down both *mad2* and a selected candidate (e.g. *asp: abnormal spindle*) we observed apoptosis in the affected area (dark staining in Fig. 2e”). Similar caspase results were seen for other candidates (data not shown). Together, these results suggest that knockdown of the candidates from our screen does not kill normal cells but does cause cell death by apoptosis in these CIN cells. This apoptosis could result if the candidate knockdowns generated CIN themselves, as high levels of CIN can be cell-lethal [36]. To test whether loss of our candidates induced apoptosis by increasing the level of CIN over a viability threshold, we tested whether their depletion induced CIN in normal cells by scoring mitotic cells from larval brains. We did not see any significant increase in anaphase errors (Figure S5), suggesting that depletion of the candidates alone does not generate CIN.

### Involvement of DNA Damage

**Figure 3:**
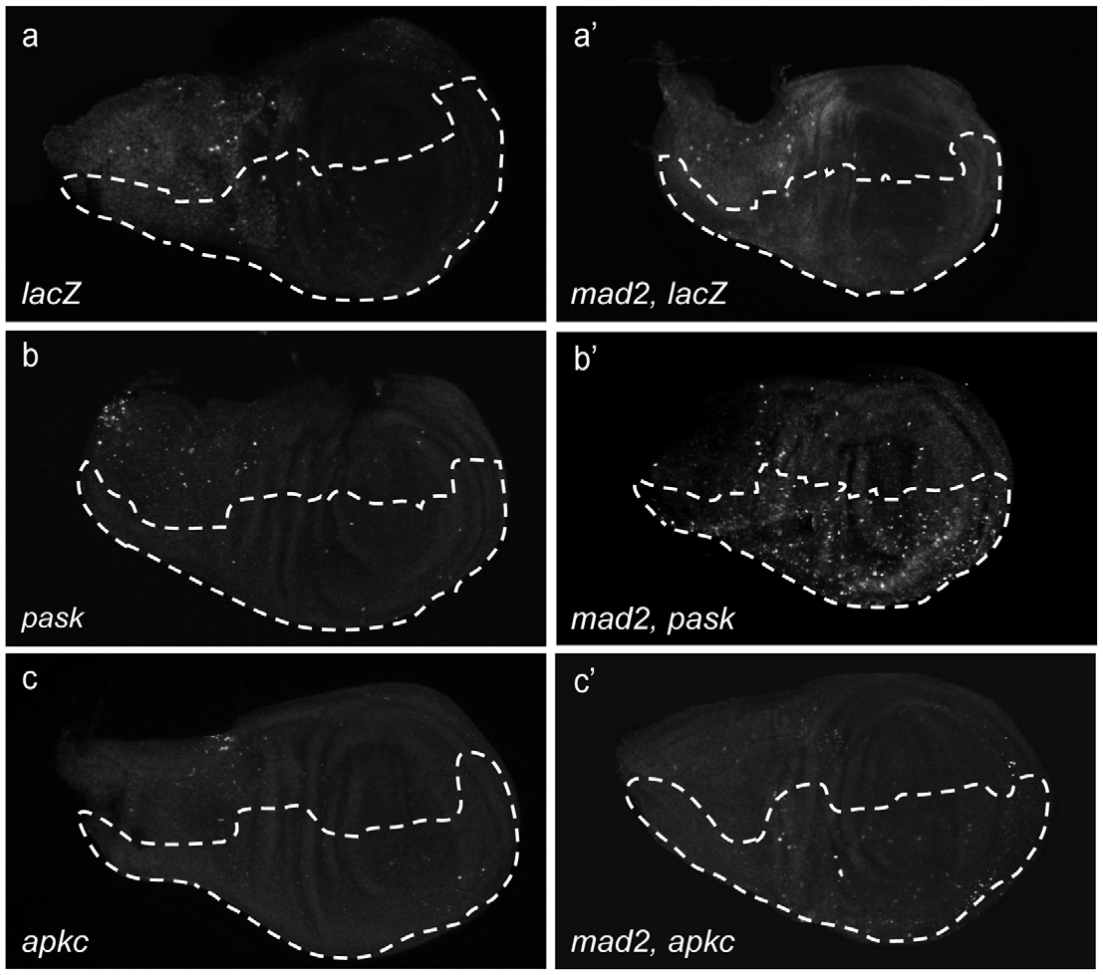
DNA damage (anti-P-H2AvD) staining of third instar larval wing discs. (a–c′) Dotted line shows the *en-CD8GFP* marked test region in which genes were depleted. The other half of each wing disc expressed no transgenes. (a, a′) Negative control (*LacZ RNAi*) with and without Mad2 (b, b′) PASK depletion with and without Mad2. (c, c′) aPKC depletion with and without Mad2. Significant induction of DNA damage in the depleted area is seen in *Pask, mad2* discs but not *LacZ, mad2* or *aPKC, mad2* discs.

Because double-stranded DNA breaks are a well known cause of anaphase errors and are implicated in *p53* dependent cell death [37], [38], we tested for DNA damage by anti-γH2aX antibody staining in knockdowns of selected candidates with and without Mad2 in larval wing discs (Figure 3). Depletion of *Pask, bsk, loki, Nek2, CG8878, asp, mbt or CG4041* in CIN cells gave an elevated level of DNA damage, compared to the *lacZ* RNAi negative control (Figure S3). Other candidates such as *aPKC*, when depleted in CIN cells, did not show a significantly elevated level of DNA damage as compared to the negative control (Figure S3), although they showed a high level of apoptosis (Figure S2A). In contrast, DNA damage in *Pask* (PAS kinase) depleted CIN cells was significantly higher than *aPKC* and *Nek2* (Figure 3 and S3), although *Pask* showed lower levels of apoptosis. Our results show that the CIN dependent apoptosis generated by candidate depletion was often, but not always, associated with an increase in double stranded DNA breaks.

### P53 Dependence for Induced Cell Death in CIN Cells

**Figure 4:**
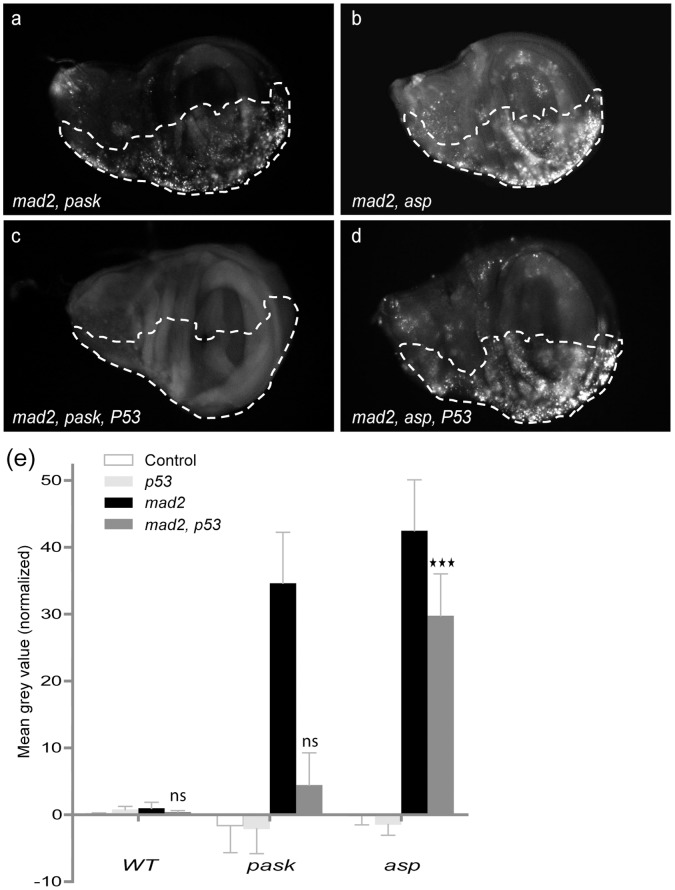
P53-dependent and p53-independent apoptosis. (a–d) Dotted line shows the *en>CD8GFP* marked test region and the other half expressed no transgenes. Acridine Orange staining on double (a–b: *Candidate* and *mad2*) and triple knockdown (c–d: *Candidate*, *mad2* and *p53*) wing discs. (e) Graph shows quantitation of Acridine Orange staining (above wild type) in control and candidate knocked down imaginal wing disc halves. The first bar of each group represents *candidate RNAi* alone (control), the second bar represents *candidate RNAi with p53RNAi* (P53), the third or black bar represents *Candidate* and *mad2* knocked down and the fourth bar represents triple knockdown (*Candidate*, *mad2* and *p53*). Error bars represent 95%CIs, n≥8 in all cases. P-values are calculated by two-tailed t-tests with Welch’s correction: p<0.001 = ★★★ and p>0.05 = ns (not significant). Tests compare *candidate mad2 p53* with *candidate* alone to test whether significant *p53*-independent cell death is seen when each candidate is co-depleted with Mad2. Significant levels of *p53*-independent cell death are seen for *asp, mad2* but not *Pask, mad2.*

Because P53 is commonly lost in tumours and has been implicated in their CIN tolerance [21], [22], we tested the effect of P53 knockdown in several candidates from our screen. Specifically, we examined whether loss of P53 affected the ability of our candidates to induce cell death in a CIN background. Depletion of P53 suppressed the loss of tissue phenotype when *Pask* and *mad2* were both depleted in adult wings (data not shown) and significantly decreased the level of apoptosis in wing discs (Figure 4), suggesting that the cell death in this case was largely *p53* dependent. However for *asp*, depleting P53 had little effect in the double knockdown (*asp* and *mad2*) wing discs, showing that, in this case, the apoptosis induced by *asp* knockdown in CIN cells is largely P53 independent (Figure 4). Other candidates such as *bsk* (Jun kinase) gave a modest reduction in the level of cell death when P53 was depleted (data not shown), suggesting the involvement of both *p53* dependent and independent mechanisms in inducing cell death in this case.

## Discussion

To identify targets that could be depleted to induce apoptosis in cells with chromosomal instability, we carried out RNAi screening of kinases and phosphatases in a CIN model system. We targeted CIN because it is common in cancers and CIN makes these cancers resistant to current therapies [3]. Using *Drosophila* as a model, we were able to induce CIN in a genetically stable background by depleting ∼85% of Mad2, which weakens SAC function and shortens metaphase [25], [39]. We found this gives an optimal level of CIN that is enough to screen against, but not so much that cells cannot survive. This approach has some advantages over using vertebrate CIN cell lines [40] which, by definition, have highly diverse and unstable genomes. In particular, we were able to test each candidate on genetically identical control tissues, which allowed us to be confident that any apoptosis was due to the effect of the checkpoint defect, not an artifact of a particular aberrant cell line.

The screen identified a significant group of candidates, including Nek2, JNK and Asp, that are directly or indirectly linked to the centrosome (Table 1). The centrosome is a regulatory hub of the major events during mitosis: alterations in centrosomal proteins and numbers result in segregation defects and CIN [41]. Extra centrosomes are common in cancer, and contribute to CIN by forming multi-polar spindles, which produce more merotely and lagging chromosomes and hence whole chromosome aneuploidy [42], [43]. Our identification of Nek2, which is currently being pursued as therapeutic target for cancer [34], confirms that our screen has the potential to identify clinically significant drug targets for CIN tumors.

Members of the JNK pathway, which is known to promote apoptosis, DNA damage response, proliferation, migration and differentiation [44]–[46] were also found in our screen. Our results suggest a novel role for JNK in preventing cell death in response to mitotic errors. This may potentially explain the anti-apoptotic effect of JNK seen in HCC tumours and leukemia [44].

Abnormal spindle (Asp) binds at the minus end of microtubules, and is required for centrosome attachment [47] and possibly for down-regulating p53 [48]. In this respect it is interesting that we observed p53 independent cell death in *asp mad2* double depletions. This suggests that the apoptosis we observed was not simply a result of losing a negative regulator of p53. Further work will be required to determine what triggers cell death in this case.

Taken together, these results suggest that CIN cells are highly sensitive to centrosome disruption, responding by apoptosis to treatments that have no effect on normal dividing cells. One plausible hypothesis to explain this sensitivity was that the centrosome disruption by itself caused a certain rate of CIN, which when added to the CIN from Mad2 depletion, took the cells over a threshold of instability beyond which they were unviable [36]. Our data do not support this model, as we did not detect a significant rate of anaphase errors when any of our centrosomal candidates were depleted by themselves (Figure S5). An alternative explanation is that there is significant crosstalk between events at the centrosome and events at the kinetochore that renders SAC-deficient cells particularly dependent on centrosomal signals. This dependence may relate to the centrosomal localization of p53 [48], [49], DNA damage repair proteins [50] or even Mad2 itself [51].

Several interesting candidates identified in our screen, however, are not localized on centrosomes (e.g. Pask and aPKC). Furthermore, some candidates have no reported connection of any kind with mitosis, indicating that we may have detected novel pathways that sense segregation defects and provide stability to cancer cells against CIN. For example, Pask is a serine/threonine kinase involved in sensing and regulating cellular energy homeostasis [52], [53]. Here we show its novel role in preventing DNA damage and p53 dependent apoptosis in CIN cells. Our screen also isolated candidates that are involved in the DNA damage response (*tefu, lok, bsk*, and *pp1a*), suggesting a role for the DNA damage response pathway in responding to CIN. This is not surprising given the recent work showing that anaphase errors result in DNA damage [36], [54] and the role of Mad2 in delaying anaphase onset to give time for repair [55].

It seems clear that the DNA damage we observed in our candidates in the presence of CIN is not simply part of the apoptotic program: we see strong apoptosis and little DNA damage in *aPKC* knockdown in CIN cells. Furthermore our highest levels of DNA damage were seen in *Pask mad2* double depletions, which gave no more apoptosis than *aPKC.* It is striking that none of our candidates alone, nor *mad2* alone, gives significant levels of DNA damage. Our interpretation of this is that the cellular DNA damage response can keep the level of damage below our detection sensitivity in any of the single depletions, and it is only when multiple checkpoint and repair mechanisms are depleted that we see unrepaired damage and widespread apoptosis.

Depletion of Asp or Bsk gives some P53 independent apoptosis in CIN cells, which could make them desirable therapeutic candidates in a clinical setting, where P53 is often absent [56], and indeed JNK inhibitors are currently in clinical trials [57]. Unfortunately, JNK is involved in many processes that make it problematic as a drug target [58]. However, the other candidates that regulate JNK signaling, (Hep: *hemipterous/*JNKK, Slpr: *slipper*/JNKKK, Pak3/Pak2 and Pvr: *PDGF/VEGF receptor*), may have potential as good CIN-specific targets.

In summary, we have used a new model for CIN in *Drosophila* to demonstrate the principle that it is possible to selectively kill CIN cells. Our RNAi knockdown identified candidates not previously known to have mitotic roles but whose depletion has the potential to kill proliferating CIN cells. Further characterization of screened candidates and their pathways may help to identify mechanisms by which cancer cells can tolerate the adverse effects of CIN and aneuploidy which in turn may lead to the identification of novel targets that can specifically kill advanced, drug resistant, CIN tumor cells without harming normal cells.

## Materials and Methods

### Stocks

All UAS-RNAi lines used, including 485 UAS- *Candidate RN*Ai (kinase and phosphatase) lines, were obtained from the Vienna *Drosophila* RNAi Centre. The *mad2-RNAi* line used was v47918. Ubiquitous expression was driven using *daughterless* (*da*)*-Gal4* and posterior wing disc expression was driven using *engrailed* (*en*)- or *hedgehog* (*hh*)*-Gal4* (Bloomington stock center).

### Screening

In viability screening we have tested ubiquitous *(da>Gal4)* knockdown of candidates (the kinase and phosphatase genes, see Table S1) in the CIN background (*mad2 RNAi*): UAS>*mad*2-RNAi/CyO; *da*>*Gal4*/TM6 *tub>Gal80ts* × *UAS> candidate*-RNAi. The temperature of crosses was adjusted to give the best numbers of progeny while still getting effective knockdown (mostly 23°C). The ratio of the average viable fly count of double knockdown (*mad2* and *candidate*) over single knockdown (*candidate* only) progeny gave the level of viability of that candidate in CIN flies. Depletion of candidates that gave >75% lethality when *mad2* was co-depleted, compared to the candidate knockdown alone, were retested and, if reproducible, considered for further assays. This was measured by counting the number of surviving Cy versus non-Cy progeny from each cross, selecting those with a ratio of at least 4∶1.

### RNA Purification and Quantitative Real-time PCR (qPCR) Assays

For each genotype, thirty brains or sixty imaginal wing discs from third instar larvae were dissected in 1X PBS and quickly transferred and homogenized into pre-cooled Trizol reagent (Invitrogen). This was done in three biological replicates. Samples were then frozen in liquid nitrogen and kept in −80 C until RNA extraction. Total RNA was extracted with chloroform and precipitated with ethanol. RNA was further purified using the RNeasy Mini Kit (Qiagen).

The qPCR assays were carried out using protocols described in [59]. Each reaction was done in triplicate for all biological replicates. Superscript III (Invitrogen) was used to make cDNA and the relative quantitation was done by using the SYBR Green mix and ABI Prism 7000 Sequence Detection System (Applied Biosystems). The mRNA levels were normalized by the mRNA levels of house-keeping gene ribosomal protein 49 (rp49) from *Drosophila*.

### Primers Pairs for Drosophila Mad2, Pask and Bsk qPCR Assay

*mad2 F/R:* GGCGACCAAAAACTGCATCA/GGTAAATTCCGCGTTGGAAGA, *bsk F/R:* GAATAGTATGCGCCGCTTACGA/ATTCCCTATATGCTCGCTTGGC, *asp F/R:* AGGCAAAGGCGGTAAACTCTGT/ACTCCGAACACCACATGAGCAG and (House-keeping gene) *rp49 F/R*: ATCGATATGCTAAGCTGTCGCAC/TGTCGATACCCTTGGGCTTG [59].

### Histology

Loss of tissue in the posterior compartment of adult wings was scored by measuring the distance from where the fifth vein met the margin, to the fourth vein. Wings of adult females were dissected in ethanol and mounted in Aqua Poly/Mount (Polysciences, Inc.). Levels of CIN were tested by counting defective anaphases in fixed, Hoechst 33342 stained brain squashes as described [60]. Briefly, third instar larval brains were dissected in PBS, fixed for 30 minutes in 4% formaldehyde, then treated with 45% acetate for 30 seconds and put into a drop of 60% acetate for 3 minutes before squashing, freezing in liquid nitrogen and leaving in ethanol until staining with 5 ug/ml Hoechst 33342 in PBS plus 0.2% Tween20, then washing with PBST and mounting in 80% glycerol. All clear anaphases in each brain were photographed and scored as normal or defective. Defective included those with bridges, broken bridges or lagging chromosomes. Slides were coded and scored without knowing their genotype.

Cell death in 3rd instar larval imaginal wing discs was measured using a vital stain (Acridine Orange). Third instar larvae were selected and dissected, and imaginal discs were collected carefully in PBS. Imaginal wing discs were then incubated for 2 min in a 1 µM Acridine Orange solution and briefly rinsed in PBS before immediately mounting and imaging. The discs were transferred to a slide having double sided sticky tape on either side of the sample. This was done to prevent the squashing of discs when we placed a cover slip on top. Acridine staining was normalized by subtracting the mean intensity value of the wild type anterior compartment from the mean intensity value of the mutant posterior compartment (marked with *en>CD8GFP*), using ImageJ software. Before normalization, background noise was subtracted from all the images by setting the rolling ball radius at 10 pixels.

Cleaved caspase 3 immunostaining was performed on dissected wing imaginal discs. Single and double knockdown third instar larvae were selected and dissected in PBS and fixed in 4% formaldehyde for 20 minutes. Fixed discs were extensively washed with PBST (1xPBS+0.2% Tween) and then blocked by PBST containing 5% fetal calf serum (PBSTF) for 30 minutes. Discs were then incubated in primary antibody solution (1∶100 Cleaved Caspase 3 Antibody from Cell Signaling in PBSTF) for 2.5 hrs at room temp or left overnight at 4°C followed by 2–3 quick washes with PBSTF and then 30 minutes in PBSTF. For secondary antibody staining, discs were incubated for 2 hrs in the dark at room temperature with 1∶75 Anti rabbit CY-3 Antibody from Abacus/Jackson in PBST followed by 2–3 quick washes with PBST and then 30 minutes incubation in PBST. Mounting was done with 80% glycerol-PBS. DNA damage staining was done using the same method with rabbit anti-H2AvD P-Ser137 (1∶700; Rockland) which is the *Drosophila* equivalent of vertebrate γH2aX, and anti-rabbit Cy3-conjugated secondary antibody (1∶100; Abacus/Jackson). Quantitation of DNA damage staining *(anti-P-H2AvD)* was also done on normalized mean intensity value. Normalization was done by subtracting the mean intensity value of the wild type anterior compartment from the mean intensity value of the mutant posterior compartment (marked with *en>CD8GFP*), using ImageJ software. Before normalization, background noise was subtracted from all the images by setting the rolling ball radius at 5 pixels. All microscopy was done on a Zeiss Axioplan2 with Semrock Brightline filters and measurements and quantitation were done using Axiovision software (Carl Zeiss). Images were compiled using Axiovision (Carl Zeiss), Photoshop and Illustrator (Adobe) software.

## Supporting Information

Figure S1 **a. Loss of tissue in adult wings.** *Engrailed*-driven single (*candidate-RNAi* only) and double (*candidate* and *mad2-RNAi*) knockdown in adult *Drosophila* wings. This driver depleted genes only in the posterior compartment of the wing, the lower half of each wing in this figure. We measured the loss of tissue by measuring the shortest distance from where the fifth longitudinal vein met the margin, to the fourth longitudinal vein (arrows). Depletion of *Pask* shows posterior wing margin notching (arrowheads) along with shorter inter-vein distance (see Figure S1b), when co-depleted with *mad2* RNAi. ***Figure S1 b:*** *Quantification of loss of tissue in adult wings:* Graph shows the average distance between 4th and 5th vein of adult wings as in S1a, which measures loss of tissue in the *engrailed* test region. Light grey bars represent *candidate RNAi* alone, dark grey bars show double knockdowns (*candidate RNAi* with *mad2 RNAi*). Negative control (*W^1118^*) showed no significant tissue loss with (black bar) or without CIN. The Y-axis starts from 250 µm, to improve resolution. dWNK was an outlier not included in this graph, showing an inter-vein distance without and with *mad2 RNAi* of 245 µm and 60 µm respectively. Error bars represent 95%CIs, n≥8 in all cases.
(PDF)

Figure S2 **a:Quantitation of Acridine Orange staining on larval wing discs.** Graph shows quantitation of Acridine Orange staining of control and candidate-RNAi imaginal wing discs. Quantification shows arbitrary grey value units normalized by subtracting the mean grey value of the wild type (anterior) region from the mean grey value of the affected (posterior) region for each disc. Negative control (*LacZ RNAi)* and *candidate RNAi* alone are represented in light grey bars and the double knockdowns of candidates with *mad2* are represented by dark grey bars, while double knockdown of *mad2* with the *LacZ* negative control is shown in black. Error bars indicate 95%CIs, n≥8 in all cases. P-values are calculated by two-tailed t-tests with Welch’s correction: p<0.001 = ★★★, p 0.001−0.01 = ★★, p 0.01−0.05 = ★. All t-tests compare *candidate-RNAi mad2-RNAi* with *lacZ-RNAi mad2-RNAi*. **Figure S2. (b1–b4):Acridine Orange staining on larval wing discs (complete data).** All wing discs are stained with Acridine Orange and the dotted line shows the *en>CD8GFP* marked posterior compartment or test region in which the genes were depleted. The other half of each disc expressed no transgenes. Single knockdowns of each candidate are arranged on the right and the double knockdowns with *mad2* are on the left side. Representative discs for each genotype are shown; the level of variation for each genotype can be seen in Figure S2A.
(PDF)

Figure S3 **DNA damage staining quantitation.** Graph shows a quantitative analysis of DNA damage (anti-P-H2AvD staining). The Y-axis represents the level of P-H2AvD staining in the affected half normalized by subtracting the level in the control half for each disc. Light grey bars represent the candidate knockdown in wild type background and dark grey bars represent the double (*candidate* and *mad2*) knockdown. Error bars indicate 95%CIs, n≥8 in all cases. P-values are calculated by two-tailed t-tests with Welch’s correction: p<0.001 = ★★★, p 0.01−0.05 = ★ and p>0.05 = ns (not significant).
(TIF)

Figure S4 **Cell death assay for the validation of Mad2 results with BubR1.** Dotted line shows the *en>CD8GFP* region or tester region and the other half expresses no transgenes (a-f) Images of wing discs stained with Acridine Orange. (a) *mad2* RNAi (d) *bubR1* RNAi. (b & e) *Pask* RNAi. Double knockdowns are (c): *Pask RNAi* and *mad2 RNAi* and (f): *Pask RNAi* and *bubR1 RNAi*.
(TIF)

Figure S5 **CIN levels.** Graph represents the frequency of defective anaphases in knockdowns of LacZ, Asp, Bsk, aPKC, Pask and Mad2 in brain cells. LacZ was used as a negative RNAi control and Mad2 is used as positive control to compare the level of CIN. None of the candidates show significantly elevated levels of CIN above the LacZ control. Error bars show 95%CIs, n>40 in all cases. P-values are calculated by two-tailed Fisher’s exact test: p<0.001 = ★★★ (extremely significant) and p>0.05 = ns (not significant).
(TIF)

Table S1 **List of kinases and phosphatases tested in the viability screening of our induced-CIN model.** Columns show: the gene identifiers; number of replicate crosses carried out; total numbers of CyO (*kinase-*RNAi only) and non-CyO (*kinase*, *mad2* RNAi) progeny; Ratio of the average number of Cy/non-Cy progeny per cross used to rank the table; and probability of finding a cross this diverged or more diverged from a 50/50 ratio (the null hypothesis), out of this number of crosses (936), purely by chance.
(XLSX)
