# Supplementary figures and images for: A Screen for Selective Killing of Cells with Chromosomal Instability Induced by a Spindle Checkpoint Defect
Source: PLoS One. 2012 Oct 15;7(10):e47447. doi: 10.1371/journal.pone.0047447 (PMC3471812; doi:10.1371/journal.pone.0047447)

a

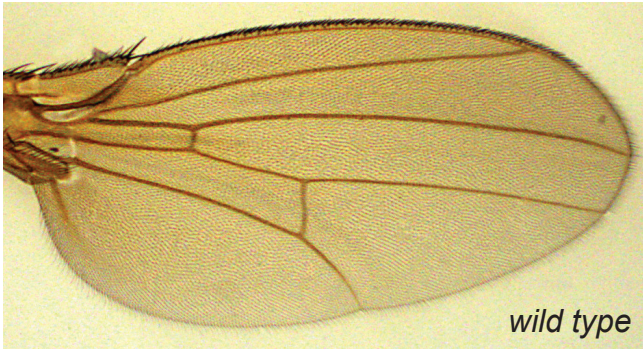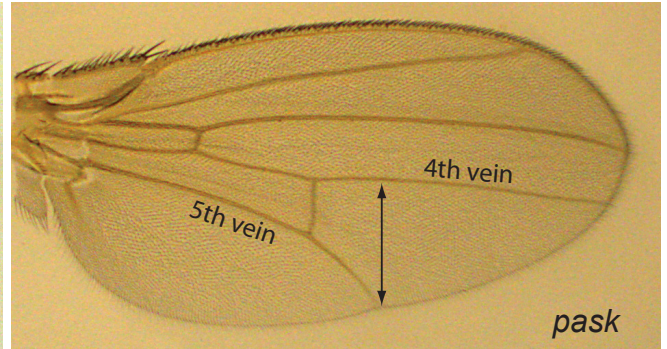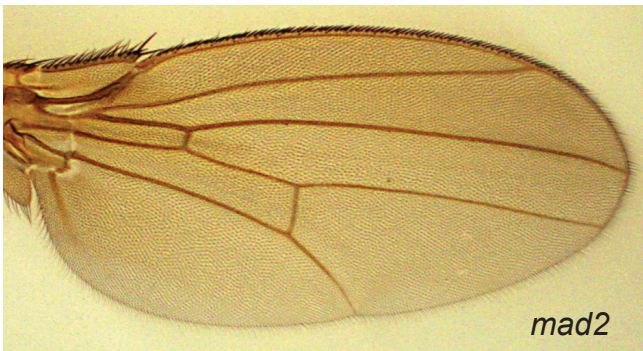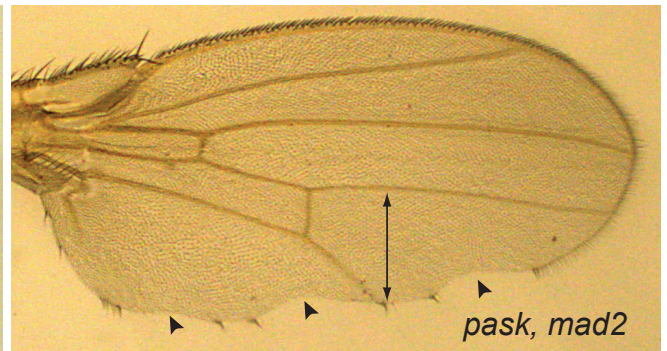

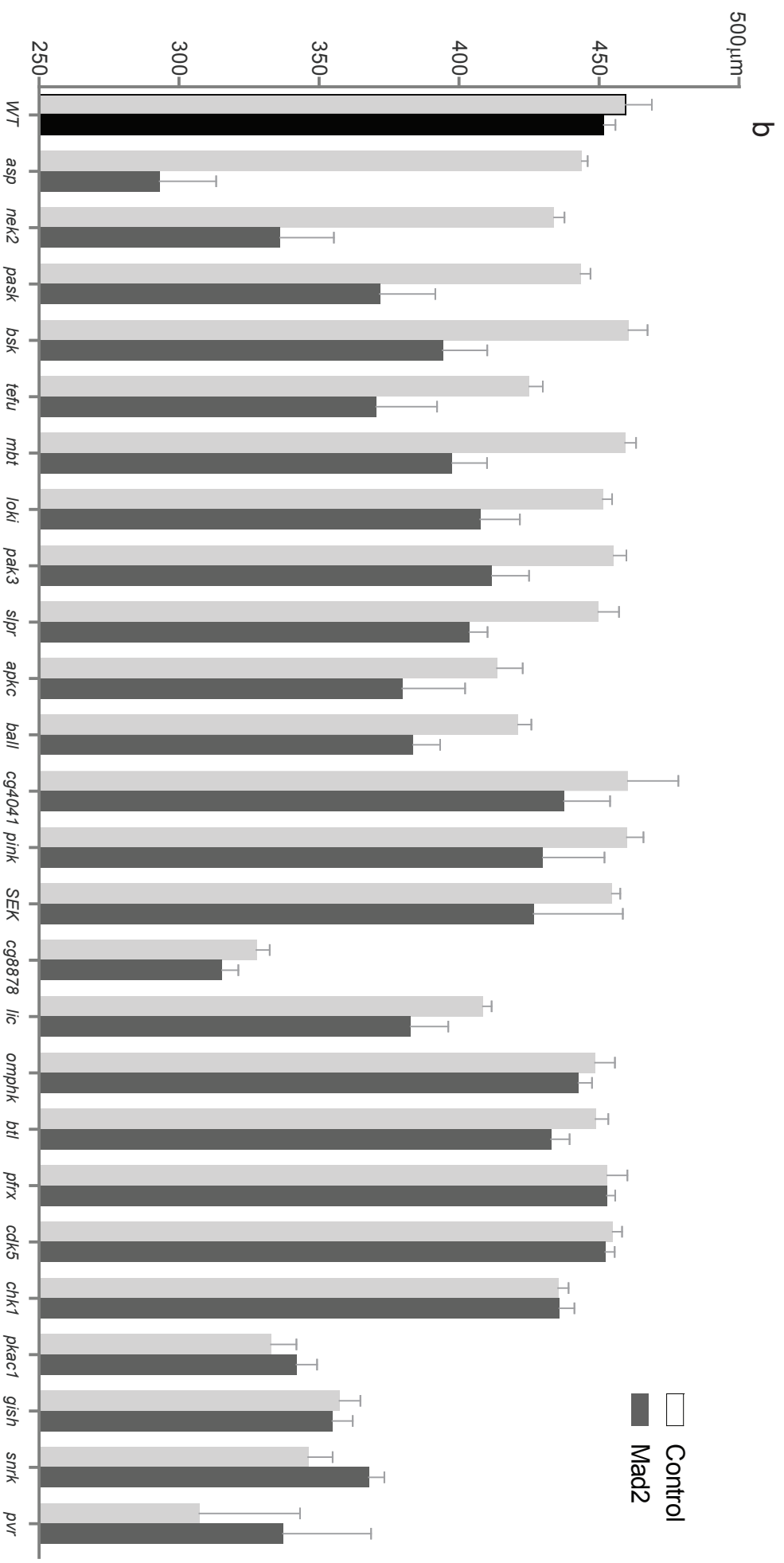

Supplement: Figure S1 — a. Loss of tissue in adult wings. Engrailed-driven single (candidate-RNAi only) and double (candidate and mad2-RNAi) knockdown in adult Drosophila wings. This driver depleted genes only in the posterior compartment of the wing, the lower half of each wing in this figure. We measured the loss of tissue by measuring the shortest distance from where the fifth longitudinal vein met the margin, to the fourth longitudinal vein (arrows). Depletion of Pask shows posterior wing margin notching (arrowheads) along with shorter inter-vein distance (see Figure S1b), when co-depleted with mad2 RNAi. Figure S1 b: Quantification of loss of tissue in adult wings: Graph shows the average distance between 4th and 5th vein of adult wings as in S1a, which measures loss of tissue in the engrailed test region. Light grey bars represent candidate RNAi alone, dark grey bars show double knockdowns (candidate RNAi with mad2 RNAi). Negative control (W1118) showed no significant tissue loss with (black bar) or without CIN. The Y-axis starts from 250 µm, to improve resolution. dWNK was an outlier not included in this graph, showing an inter-vein distance without and with mad2 RNAi of 245 µm and 60 µm respectively. Error bars represent 95%CIs, n≥8 in all cases. (PDF) [file pone.0047447.s001.pdf]

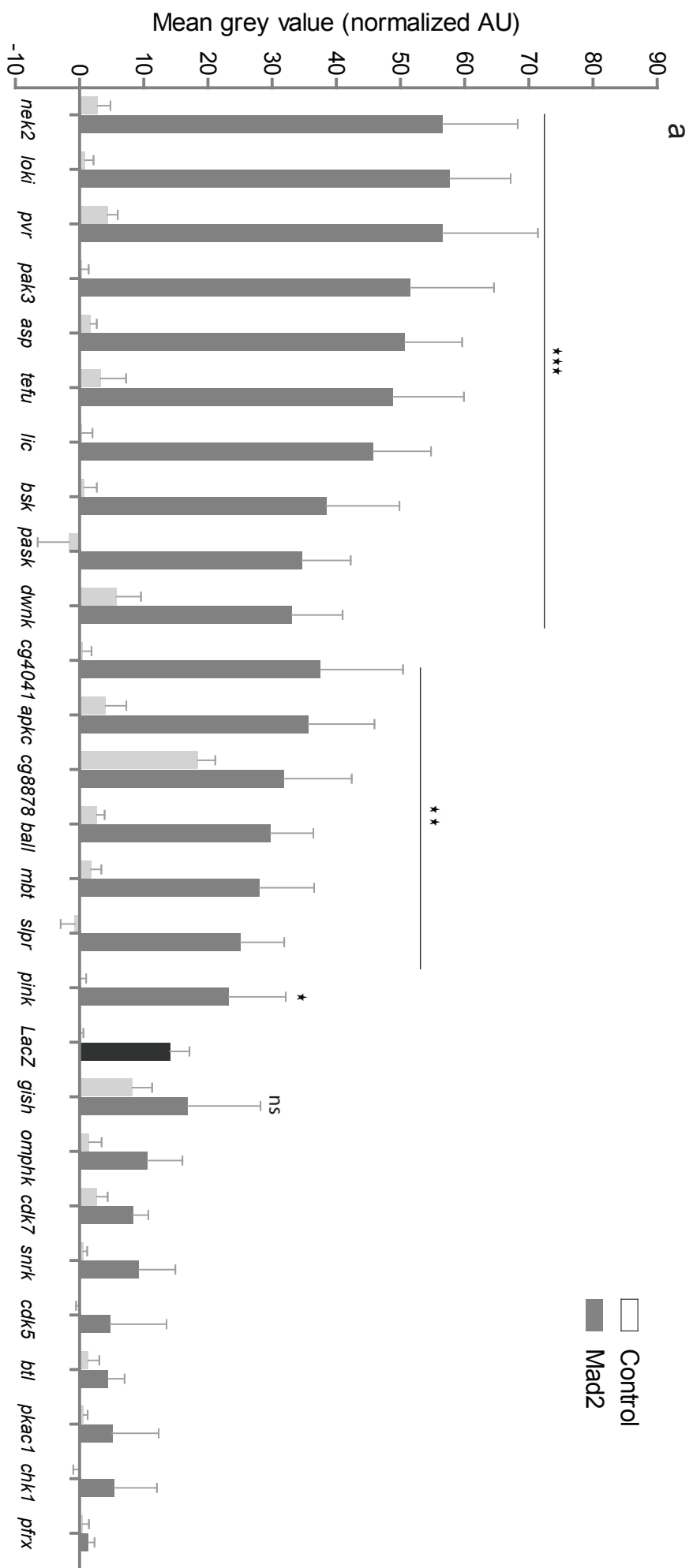

b (1)

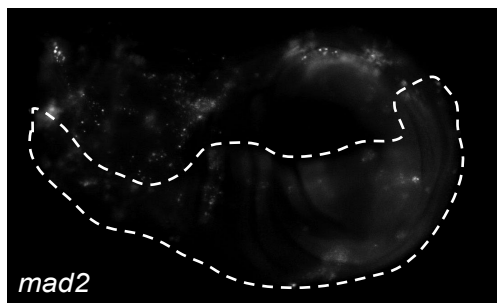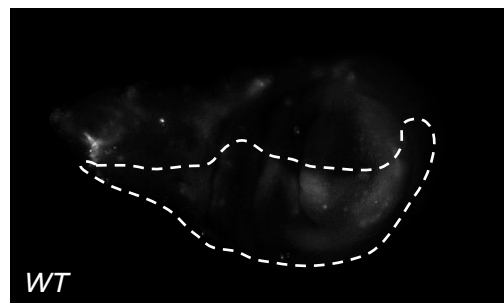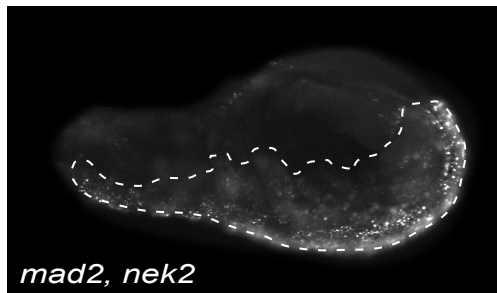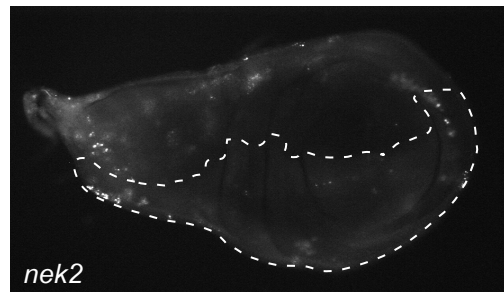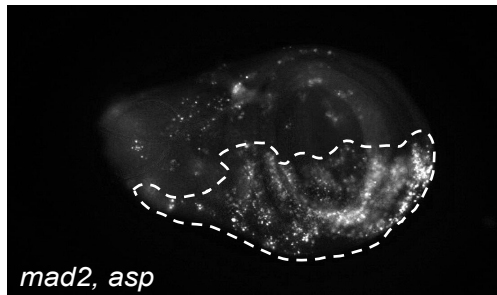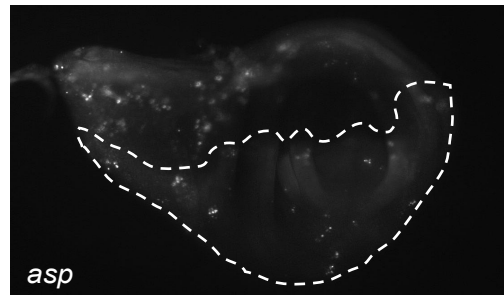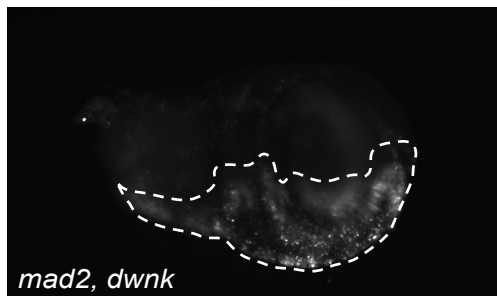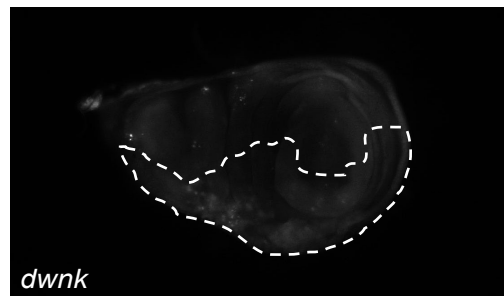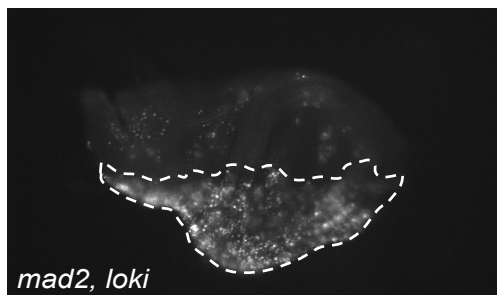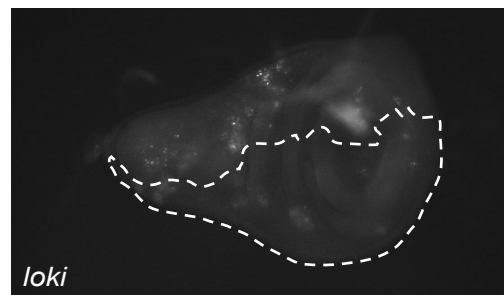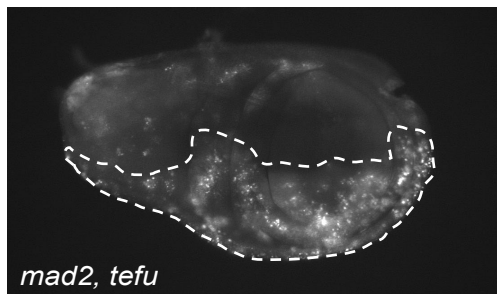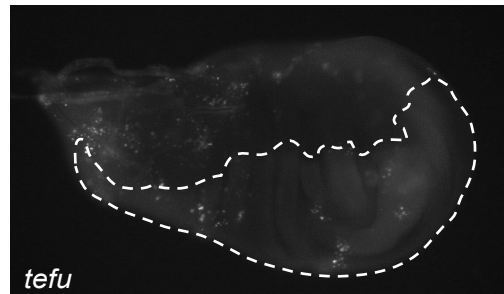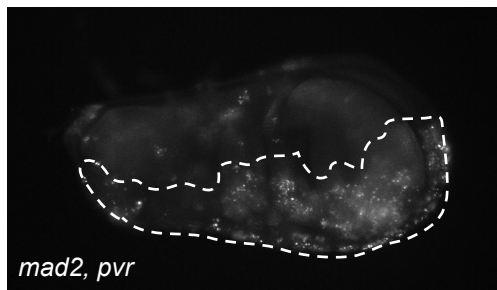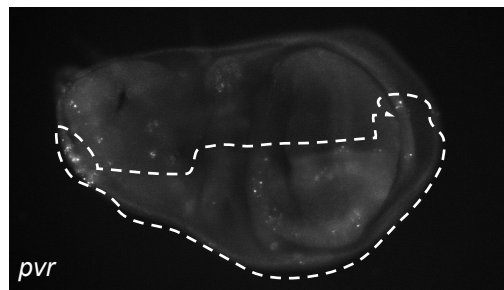

b (2)

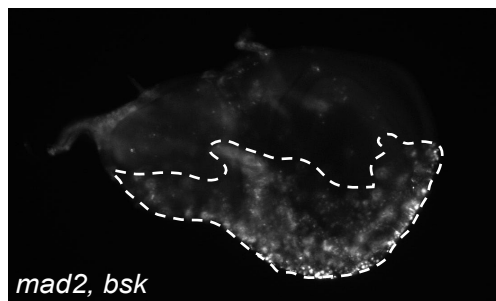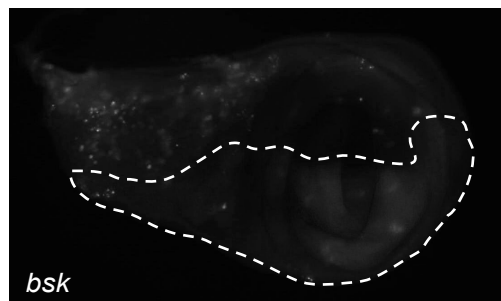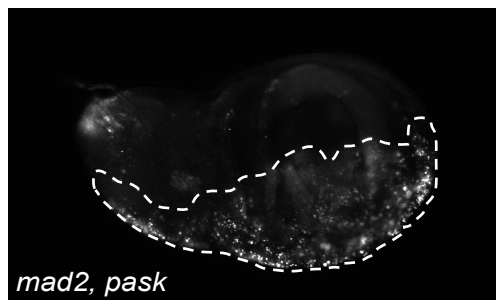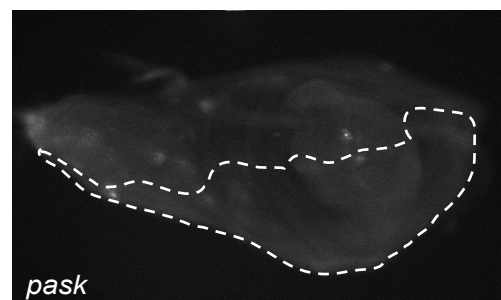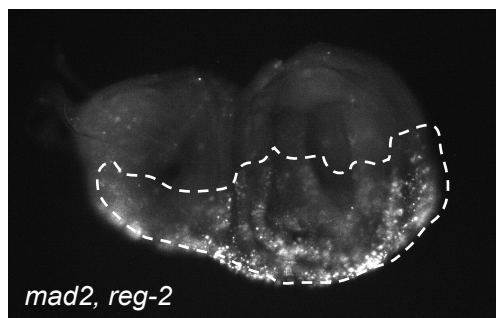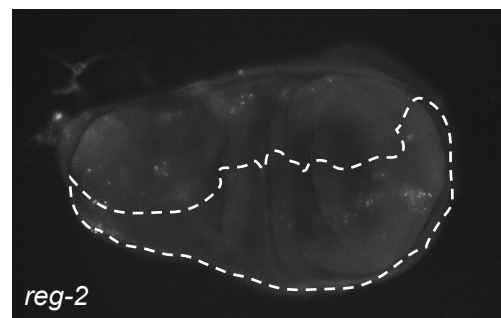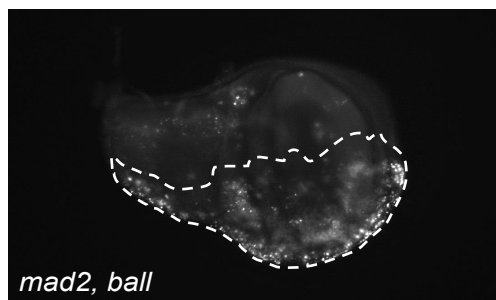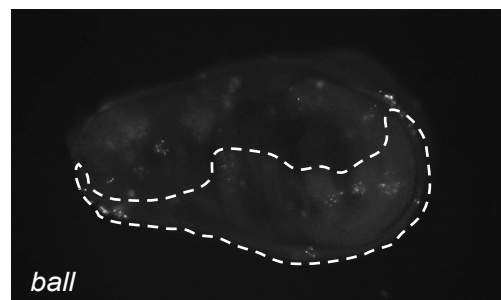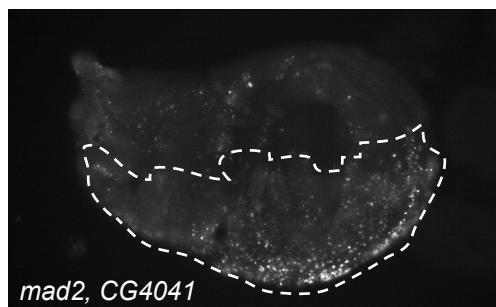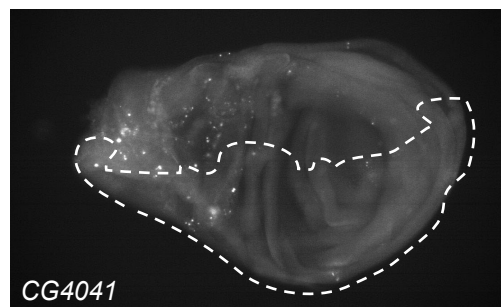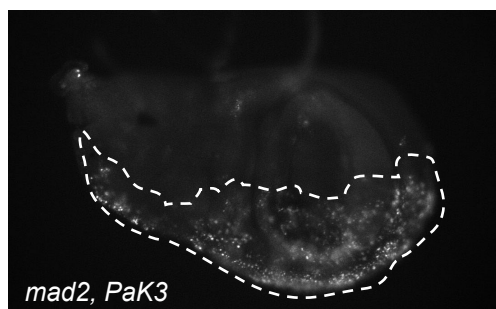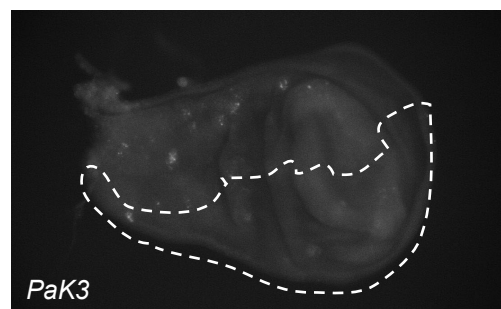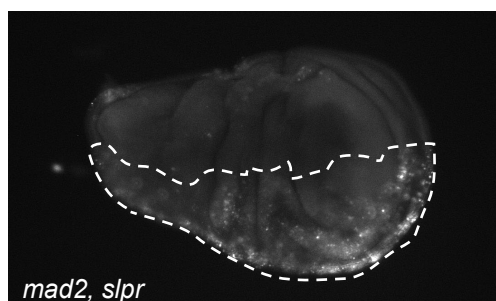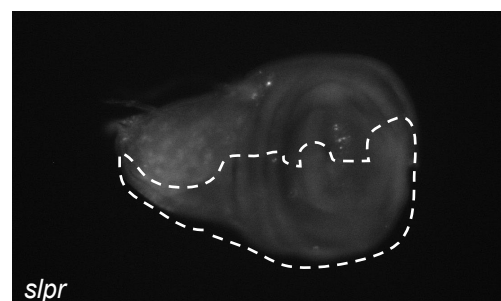

b (3)

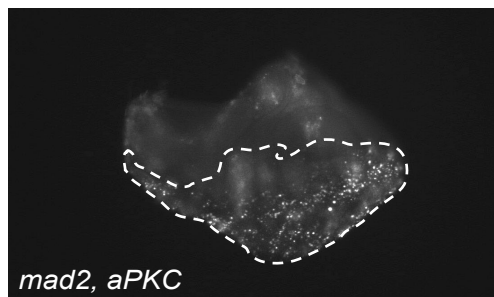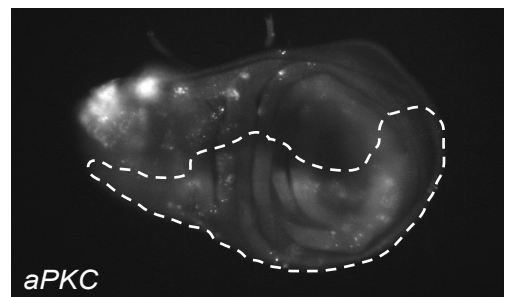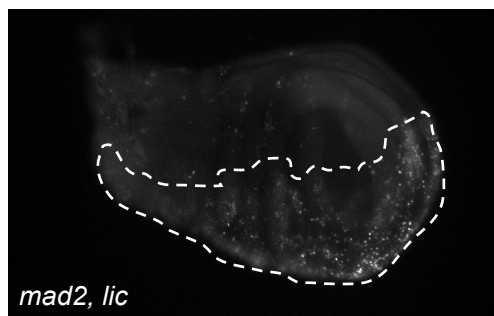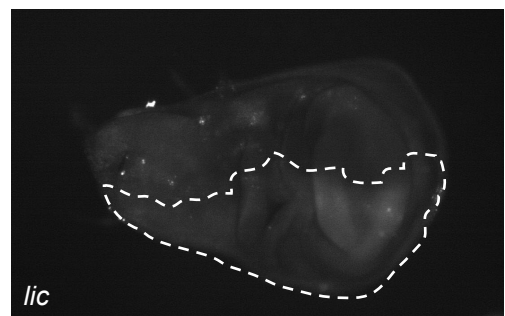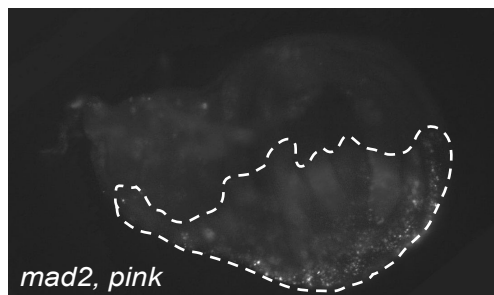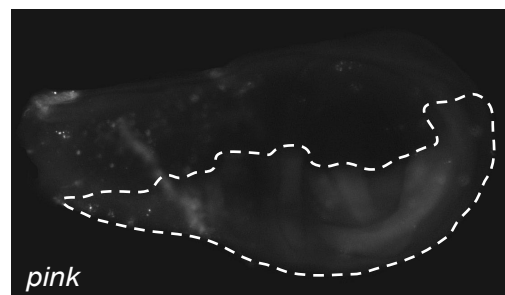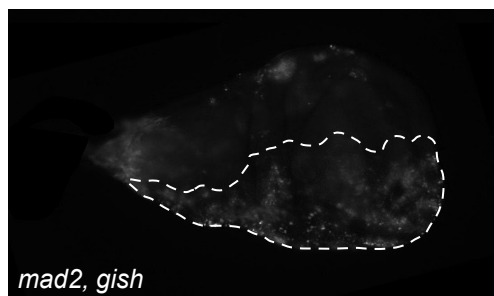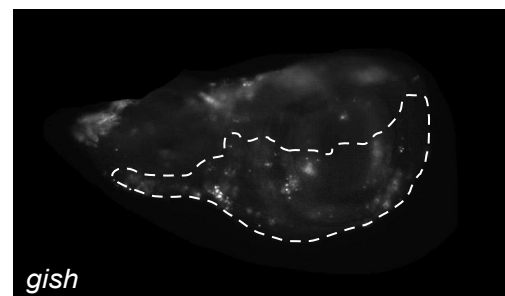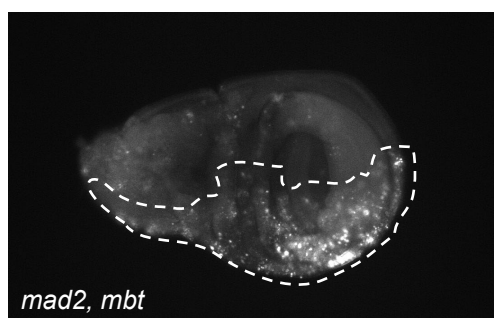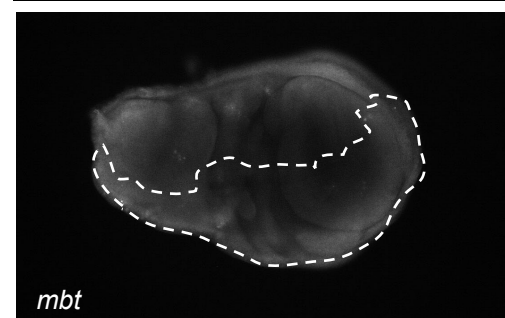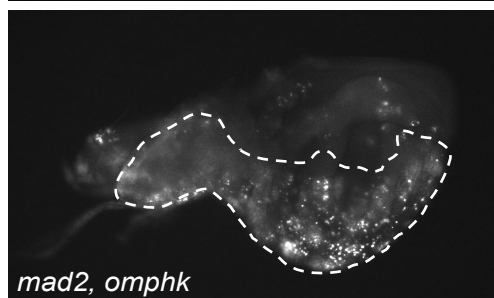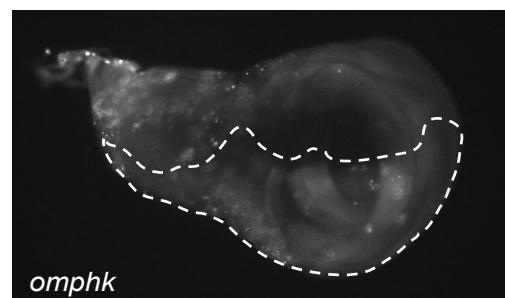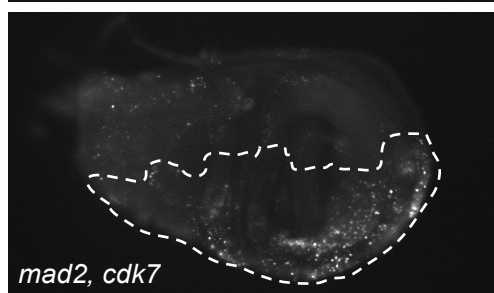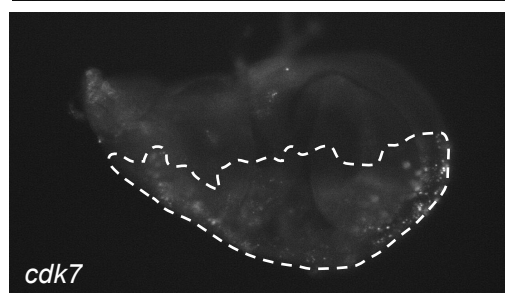

b (4)

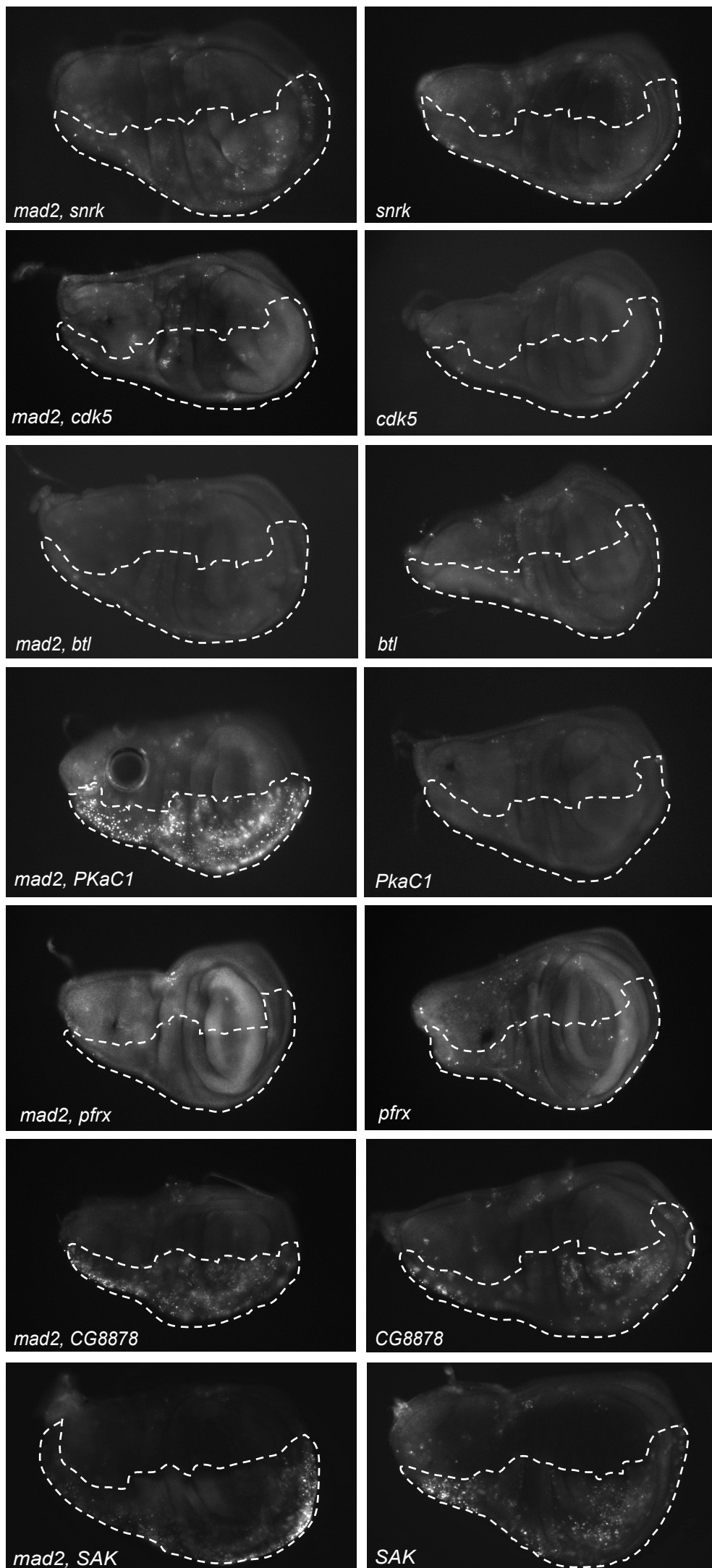

Supplement: Figure S2 — a:Quantitation of Acridine Orange staining on larval wing discs. Graph shows quantitation of Acridine Orange staining of control and candidate-RNAi imaginal wing discs. Quantification shows arbitrary grey value units normalized by subtracting the mean grey value of the wild type (anterior) region from the mean grey value of the affected (posterior) region for each disc. Negative control (LacZ RNAi) and candidate RNAi alone are represented in light grey bars and the double knockdowns of candidates with mad2 are represented by dark grey bars, while double knockdown of mad2 with the LacZ negative control is shown in black. Error bars indicate 95%CIs, n≥8 in all cases. P-values are calculated by two-tailed t-tests with Welch’s correction: p<0.001 = ★★★, p 0.001−0.01 = ★★, p 0.01−0.05 = ★. All t-tests compare candidate-RNAi mad2-RNAi with lacZ-RNAi mad2-RNAi. Figure S2. (b1–b4):Acridine Orange staining on larval wing discs (complete data). All wing discs are stained with Acridine Orange and the dotted line shows the en>CD8GFP marked posterior compartment or test region in which the genes were depleted. The other half of each disc expressed no transgenes. Single knockdowns of each candidate are arranged on the right and the double knockdowns with mad2 are on the left side. Representative discs for each genotype are shown; the level of variation for each genotype can be seen in Figure S2A. (PDF) [file pone.0047447.s002.pdf]

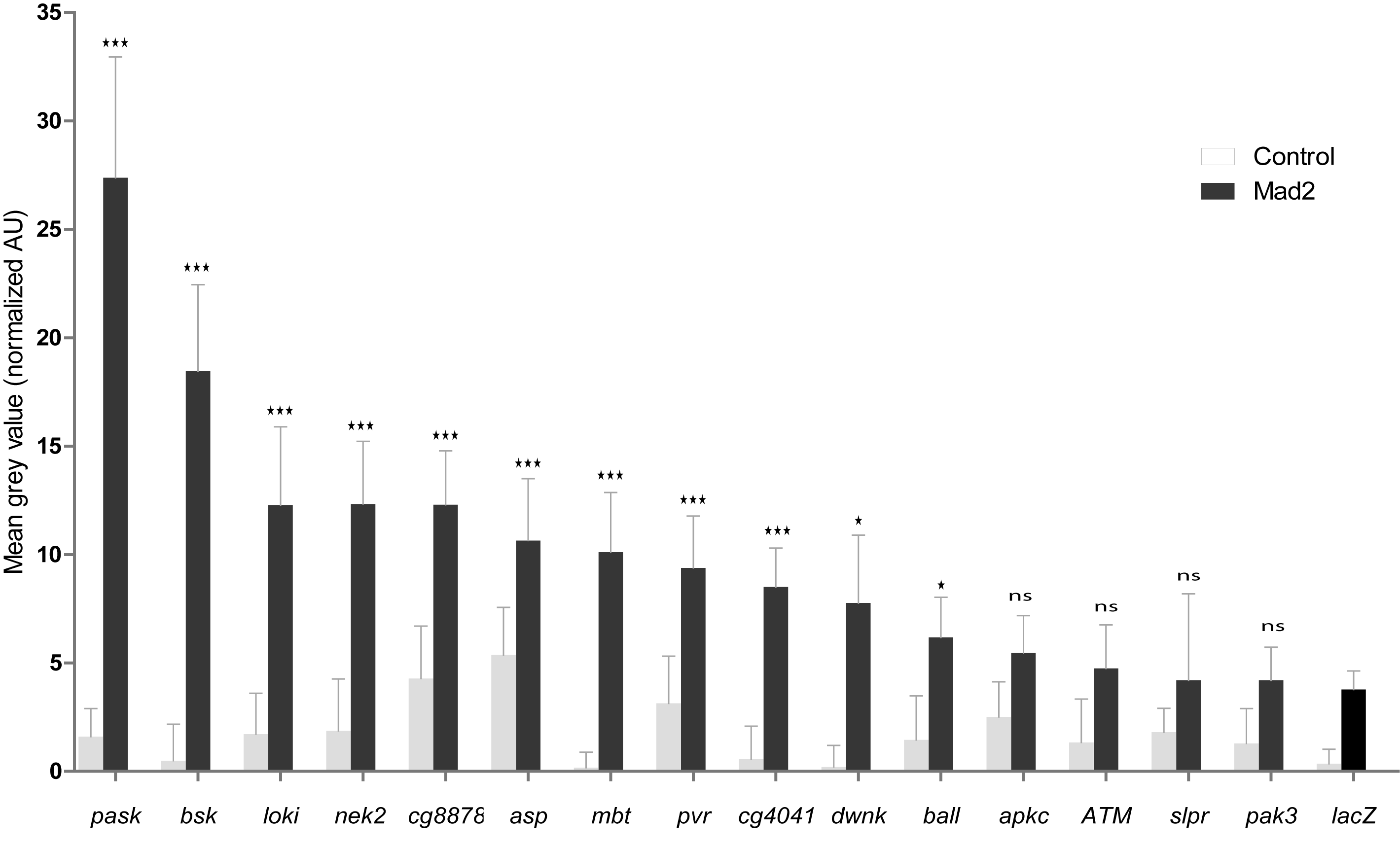

Supplement: Figure S3 — DNA damage staining quantitation. Graph shows a quantitative analysis of DNA damage (anti-P-H2AvD staining). The Y-axis represents the level of P-H2AvD staining in the affected half normalized by subtracting the level in the control half for each disc. Light grey bars represent the candidate knockdown in wild type background and dark grey bars represent the double (candidate and mad2) knockdown. Error bars indicate 95%CIs, n≥8 in all cases. P-values are calculated by two-tailed t-tests with Welch’s correction: p<0.001 = ★★★, p 0.01−0.05 = ★ and p>0.05 = ns (not significant). (TIF) [file pone.0047447.s003.tif]

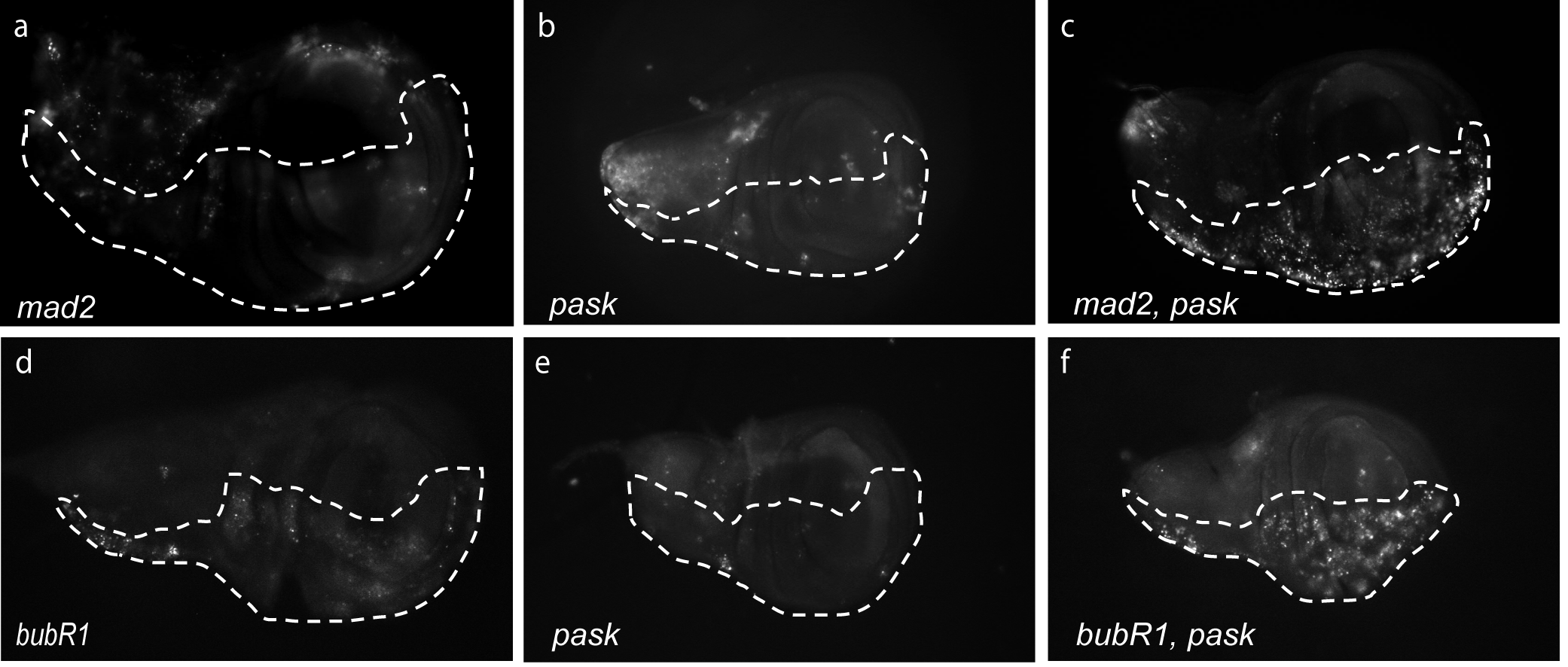

Supplement: Figure S4 — Cell death assay for the validation of Mad2 results with BubR1. Dotted line shows the en>CD8GFP region or tester region and the other half expresses no transgenes (a-f) Images of wing discs stained with Acridine Orange. (a) mad2 RNAi (d) bubR1 RNAi. (b & e) Pask RNAi. Double knockdowns are (c): Pask RNAi and mad2 RNAi and (f): Pask RNAi and bubR1 RNAi. (TIF) [file pone.0047447.s004.tif]

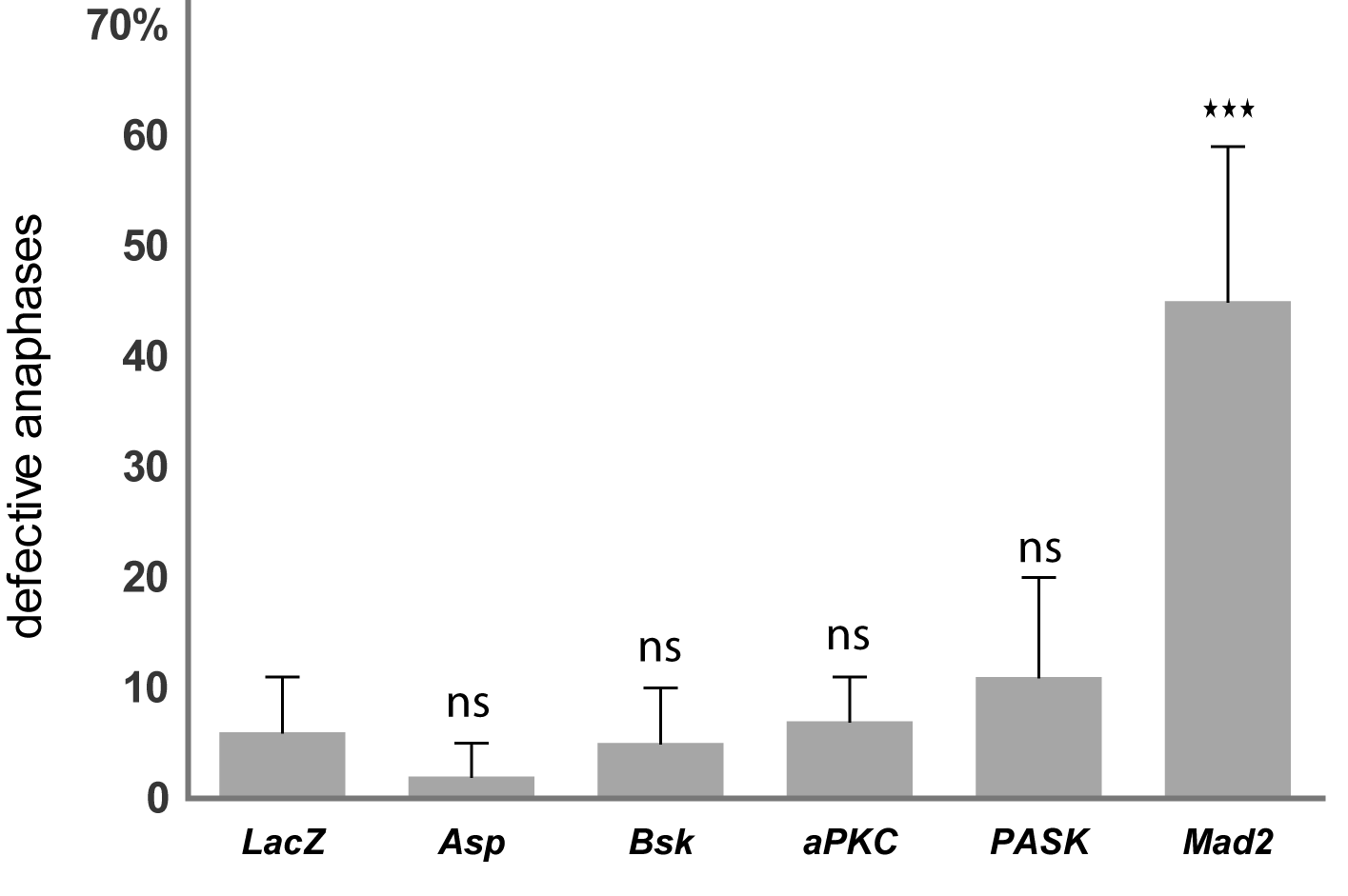

Supplement: Figure S5 — CIN levels. Graph represents the frequency of defective anaphases in knockdowns of LacZ, Asp, Bsk, aPKC, Pask and Mad2 in brain cells. LacZ was used as a negative RNAi control and Mad2 is used as positive control to compare the level of CIN. None of the candidates show significantly elevated levels of CIN above the LacZ control. Error bars show 95%CIs, n>40 in all cases. P-values are calculated by two-tailed Fisher’s exact test: p<0.001 = ★★★ (extremely significant) and p>0.05 = ns (not significant). (TIF) [file pone.0047447.s005.tif]
